# Integrating biosafety and osteogenic efficacy: a translational pathway of fluorinated porcine-derived hydroxyapatite for bone regeneration

**DOI:** 10.3389/fbioe.2026.1889739

**Published:** 2026-08-25

**Authors:** Tianze Lin, Yuanxiang Liu, Zhengying Yu, Chengwu Liu, Junming Feng, Meihua Mai, Zefeng Guo, Xinyi Yang, Yixuan Yan, Feiyang Wu, Zhiyue Wang, Mengxi Su, Lv Xie, Shiyu Wu, Zhuofan Chen

**Affiliations:** 1 Hospital of Stomatology, Sun Yat-sen University, Guangzhou, China; 2 Guanghua School of Stomatology, Sun Yat-sen University, Guangzhou, China; 3 Guangdong Provincial Key Laboratory of Stomatology, Guangzhou, China

**Keywords:** alveolar ridge augmentation, bone regeneration, bone substitute, fluorinated hydroxyapatite, long-term biosafety

## Abstract

**Introduction:**

Element doping has emerged as a promising strategy to enhance the osteogenic capacity of bone substitutes. Among various dopants, fluoride has attracted considerable attention because of its modulatory effects on bone regeneration; however, a critical gap remains regarding the long-term systemic safety of fluoride-doped materials before clinical translation. A fluoride-specific, biosafety-oriented translational pathway was therefore applied to complement conventional biomaterial assessment.

**Methods:**

Fluorinated porcine-derived hydroxyapatite (FPHA) was prepared through a chemical and thermal process and characterized to confirm the stable incorporation of fluoride into the hydroxyapatite lattice. A dual-validation dissolution assay was developed to estimate the total fluoride content of FPHA. Multiple in vitro models simulating physiological and artificial extreme environments were established to evaluate material degradation, associated fluoride release, and changes in the surrounding medium pH. A 26-week beagle dog study was conducted to evaluate serum fluoride concentrations, blood biochemical parameters, ridge contour maintenance, and bone formation.

**Results:**

Characterization confirmed stable fluoride incorporation into FPHA, which exhibited a lower degradation rate than deproteinized bovine bone mineral (DBBM) and controlled fluoride release under simulated physiological conditions. In the 26-week beagle dog study, serum fluoride concentrations remained comparable to baseline levels, with no clinically relevant adverse changes in blood biochemical parameters. FPHA also showed effective long-term ridge contour maintenance and enhanced bone formation compared to DBBM.

**Discussion:**

Collectively, these findings provide preclinical evidence supporting the preliminary long-term biosafety profile and osteogenic performance of FPHA, illustrating a fluoride-specific, biosafety-oriented evaluation strategy for ion-functionalized biomaterials.

## Graphical Abstract

**Figure:**
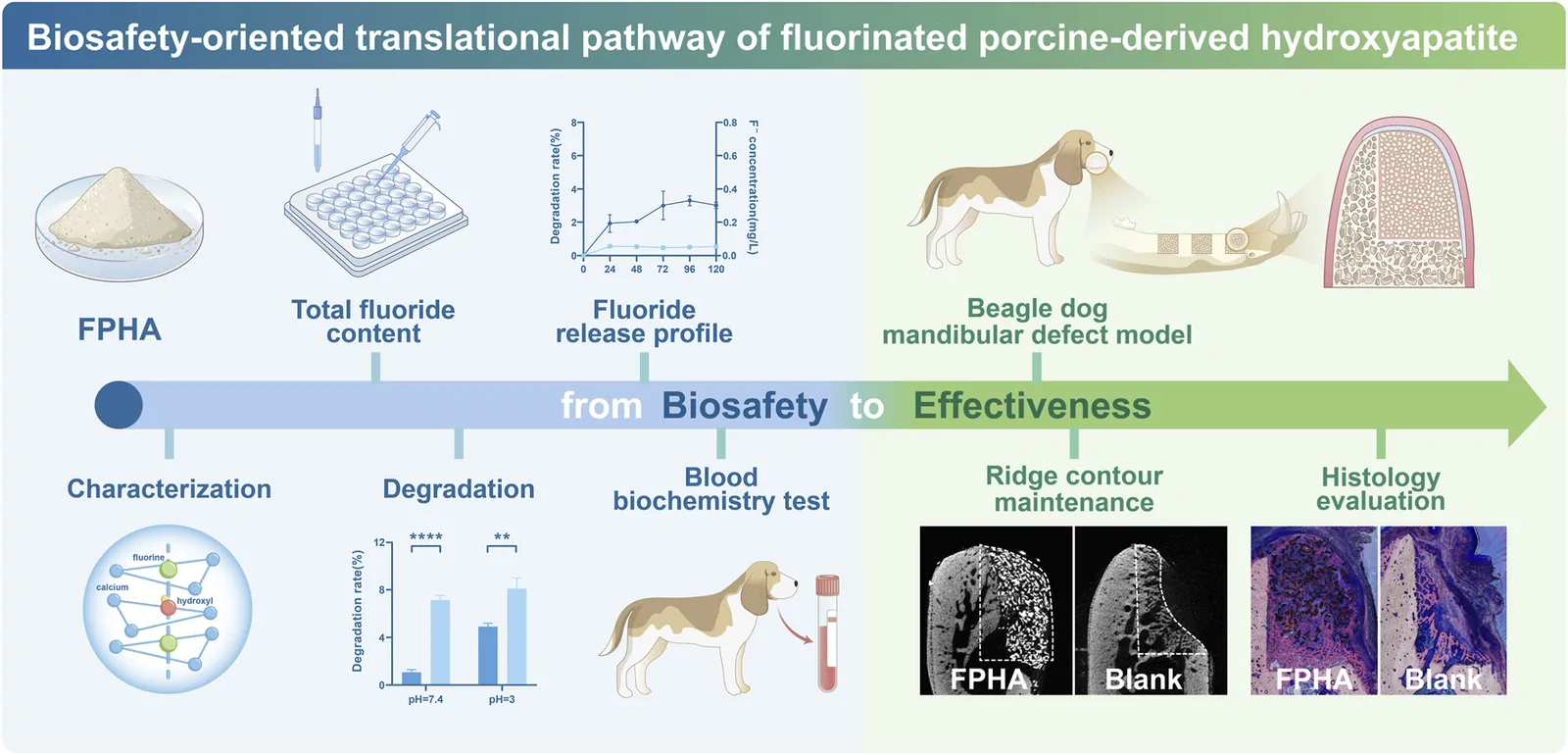
A biosafety-oriented translational pathway was established for fluorinated hydroxyapatite, demonstrating its favorable biosafety and osteogenic efficacy as a bone substitute material.

## 1 Introduction

Maxillofacial bone defects represent a major clinical challenge in periodontal and implant therapy (Sculean et al., 2015; Titsinides et al., 2019). Autogenous bone grafting has long been regarded as the gold standard for bone defect repair, with its clinical effectiveness extensively validated (Schmidt, 2021). However, the clinical application of autogenous bone grafting is constrained by limited bone availability and donor site morbidity (McKenna et al., 2022). In contrast, xenogeneic bone substitutes offer distinct advantages, including broad availability and the avoidance of donor site complications (Zhao et al., 2021). Despite these advantages, xenogeneic bone substitutes exhibit limited intrinsic bioactivity, which compromises their bone regeneration capacity (Sivolella et al., 2014; Choi et al., 2015; Ge et al., 2018; Li et al., 2026).

Accordingly, various strategies have been explored to enhance the osteogenic ability of xenogeneic bone substitutes, including surface modification, elemental doping, and loading with bioactive molecules (Lee et al., 2019; Li J. et al., 2025). Among these approaches, elemental doping has emerged as a promising strategy, owing to its multiple modulatory roles in bone regeneration and versatile delivery strategies (Luo et al., 2023): copper ion incorporation enhances osteoblastic activity and biomineralization and promotes the osteogenic and angiogenic properties of calcium phosphate materials (Lin et al., 2020; Ling et al., 2025); magnesium-containing scaffolds have shown osteogenic and angiogenic potential (Jo et al., 2024; Ling et al., 2025); strontium ions activate osteogenic signaling pathways and thereby enhance bone repair and regeneration (Cheng et al., 2023; Hu et al., 2023). Fluoride has attracted considerable attention because of its effects on apatite chemistry, mineralization, and bone formation (Dhar and Bhatnagar, 2009). Several studies have further demonstrated that fluorapatite-based scaffolds exhibit favorable physicochemical and biological properties and can support bone regeneration in preclinical defect models (He et al., 2023; Kazimierczak et al., 2023; Nielson et al., 2024). Our previous studies have suggested that fluoride ions may modulate the osteoimmune microenvironment and promote osteogenic responses (Qiao et al., 2018; Wu et al., 2019; Chen et al., 2024). Subsequent *in vivo* studies in rats, rabbits, and beagle dogs showed that fluorinated porcine-derived hydroxyapatite (FPHA), prepared through a two-step chemical and thermal process, exhibited favorable bone-regenerative performance in different defect models (Liu et al., 2018; Liu et al., 2020; Wu et al., 2024; Liu et al., 2025).

However, when considering the clinical translation from bench to bedside, biosafety must be given equal importance alongside efficacy. Assessment of osteogenic efficacy alone, without systematic biosafety evaluation, limits the clinical relevance and translational potential of biomaterials. Therefore, integrated evaluation of long-term biosafety and efficacy is essential prior to potential clinical application of biomaterials (Li Q. et al., 2025). Fluoride exhibits concentration-dependent biological effects: low concentrations may support bone regeneration (Wu et al., 2019), whereas excessive fluoride exposure may lead to adverse effects such as fluorosis (Abanto Alvarez et al., 2009). Therefore, the translational evaluation of FPHA requires a fluoride-specific, biosafety-oriented preclinical strategy that complements conventional biomaterial assessment and includes additional evaluation of material degradation, fluoride release, and potential systemic fluoride exposure.

In the present study, FPHA was evaluated through systematic assessment of fluoride incorporation, total fluoride content (TFC), material degradation, associated fluoride release, potential systemic fluoride exposure, and pH changes of the surrounding medium. A 26-week beagle dog study was conducted to monitor serum fluoride concentrations and blood biochemical parameters, while bone regeneration and residual material volume were quantitatively evaluated by micro-CT, with histological observations providing supportive evidence. The study aimed to evaluate the long-term biosafety profile and osteogenic performance of FPHA and to illustrate a biosafety-oriented evaluation strategy for ion-functionalized biomaterials.

## 2 Materials and methods

### 2.1 Preparation and characterization of PHA and FPHA

Porcine-derived hydroxyapatite (PHA) and FPHA were prepared following a chemical and thermal process as previously described (Liu et al., 2012; Qiao et al., 2017). Specifically, cancellous bone obtained from porcine femora was thoroughly cleaned, cut into small pieces, and sequentially treated with 30% hydrogen peroxide and anhydrous ethanol to remove residual organic components. The samples were then calcined in air at 800 °C for 2 h to obtain porcine-derived hydroxyapatite (PHA). Fluorinated porcine-derived hydroxyapatite (FPHA) was prepared by immersing PHA in 0.25 mol/L NaF solution at 37 °C for 24 h, followed by heat treatment in air at 700 °C for 3 h. After cooling to room temperature, the samples were thoroughly washed with deionized water, dried, ground, and sieved to obtain particles ranging from 0.25 to 1.0 mm. The FPHA particles were then packaged in glass vials and terminally sterilized by cobalt-60 gamma irradiation at a dose of 25–40 kGy. The sterilized samples were stored in sealed moisture-proof containers, and the packages were opened aseptically immediately before implantation. PHA and FPHA were prepared in three independent batches. Quantitative *in vitro* and *in vivo* experiments included samples from the independently prepared FPHA batches, whereas representative images from one batch were selected for presentation in the qualitative characterization.

The surface morphology of the obtained materials was examined by scanning electron microscopy (SEM; MIRA LM, Tescan, Czech Republic), and their elemental composition and distribution were characterized using energy-dispersive X-ray spectroscopy (EDS). X-ray diffraction (XRD; Empyrean, Malvern Panalytical, the Netherlands) was employed to analyze the crystal structure and crystallinity of FPHA. Furthermore, Rietveld refinement of the XRD patterns was carried out using TOPAS Academic software to determine the crystal structure parameters and to quantify the fraction of the fluorinated hydroxyapatite phase.

### 2.2 Determination of the total fluoride content (TFC) in FPHA

Two complementary dissolution-based methods were used to obtain cross-validated estimates of the total fluoride content (TFC) in FPHA.

Before each measurement session, a 100 ppm fluoride standard solution was serially diluted to prepare 10 and 1 ppm fluoride standards. Each standard was mixed with an equal volume of total ionic strength adjustment buffer II (TISAB II; Thermo Fisher Scientific, USA). The fluoride ion-selective electrode (Orion 9609BNWP, Thermo Fisher Scientific, USA) was calibrated using the 1 and 10 ppm standards. Calibration was accepted only when the electrode slope was within the manufacturer-recommended range of −54 to −60 mV per decade. Following calibration, the standard solutions were remeasured as calibration-verification samples, and sample measurements were initiated only after the measured standard concentrations were consistent with their nominal values.

#### 2.2.1 pH adjustment method

FPHA (350 mg) was accurately weighed and completely dissolved in 3 mL of 37% hydrochloric acid (HCl). The resulting solution was diluted to 28 mL with ultrapure water and equally divided into seven aliquots. Each aliquot was brought to a volume slightly below 15 mL with ultrapure water, adjusted to a target initial pH of approximately 0, 0.5, 1, 2, 3, 4, or 5 by the dropwise addition of sodium hydroxide (NaOH) solution, and then diluted to a final volume of exactly 15 mL. A 1-mL aliquot was collected from each pH-adjusted solution and mixed with an equal volume of TISAB II. The fluoride ion concentration was measured using the fluoride ion-selective electrode. The pH values before and after TISAB II addition were recorded as the initial pH and final buffered pH, respectively. Since the initial 350 mg FPHA was equally divided into seven aliquots, each aliquot contained 50 mg of FPHA. For each aliquot, the TFC was calculated based on the measured fluoride ion concentration and the volume (15 mL, *V* = 0.015 L), with each aliquot corresponding to 50 mg of FPHA (*m* = 50 mg) in the equation below:

$$TFC\left(\%\right)=\frac{C\times V}{m}\times 100\%$$

where *C* is the fluoride ion concentration measured in the solution (mg/L), *V* is the volume of the solution used for calculation (L), and *m* is the mass of FPHA corresponding to that solution (mg).

#### 2.2.2 Serial dilution method

FPHA (50 mg) was accurately weighed, completely dissolved in 0.5 mL of 37% HCl, and diluted with ultrapure water to a total volume of 20 mL to prepare a 40-fold diluted stock solution. Aliquots of 1.000, 0.500, 0.333, 0.250, 0.200, and 0.167 mL were independently withdrawn from the stock solution, transferred into separate tubes, and, where necessary, diluted with ultrapure water to a final volume of 1.000 mL. These aliquot-to-final-volume ratios corresponded to nominal additional dilution factors of 1, 2, 3, 4, 5, and 6, respectively, yielding overall nominal dilution factors of 40×, 80×, 120×, 160×, 200×, and 240×. Each diluted sample was mixed with an equal volume of TISAB II, and the fluoride ion concentration was measured using the fluoride ion-selective electrode. The pH values before and after TISAB II addition were recorded as the initial pH and final buffered pH, respectively.

For each dilution level, the TFC was calculated according to the following equation:

$$TFC\left(\%\right)=\frac{C\times D\times V_{0}}{m}\times 100\%$$

where *C* is the fluoride ion concentration measured in the corresponding diluted sample (mg/L), *D* is the nominal dilution factor relative to the initial 40-fold diluted stock solution (1, 2, 3, 4, 5, or 6), *V*_*0*_ is the total volume of the initial stock solution (0.020 L), and *m* is the initial mass of FPHA used to prepare the stock solution (50 mg).

### 2.3 *In vitro* degradation evaluation of FPHA

#### 2.3.1 Establishment of the degradation models

Three controlled degradation media were established to evaluate the degradation behavior of FPHA under physiological and artificial extreme conditions: (1) a physiological model consisting of 0.1 mol/L Tris-HCl buffer at pH 7.4 ± 0.2; (2) an artificial extreme model consisting of an acidic buffer at pH 3.0 ± 0.2, prepared using Tris, HCl, citric acid, and NaOH; and (3) an artificial extreme model consisting of an acidic buffer at pH 1.0 ± 0.2, prepared using HCl, KCl, and NaOH.

#### 2.3.2 Determination of the degradation rate

To evaluate the *in vitro* degradation rate, FPHA and DBBM (Bio-Oss®, Geistlich Pharma AG, Switzerland) (each 250 mg) were accurately weighed and the initial mass (*m*_*1*_) was recorded. For each material, separate samples were immersed in 5 mL of either the pH 7.4 or pH 3.0 degradation medium and incubated at 37 °C with continuous shaking at 30 rpm for 5 days. After incubation, the supernatant was removed, and the remaining materials were thoroughly rinsed, dried and weighed to obtain the final mass (*m*_*2*_). The degradation rate was calculated according to the following equation:

$$Degradation\;Rate\left(\%\right)=\frac{m_{1}-m_{2}}{m_{1}}\times 100\%$$

where *m*_*1*_ is the initial mass and *m*_*2*_ is the final mass after degradation.

### 2.4 *In vitro* evaluation of fluoride release and medium pH stability

Fluoride release under simulated physiological conditions was first evaluated using simulated body fluid (SBF; PH 1820, Phygene, China). FPHA (100 mg) was immersed in 500 μL of SBF and incubated at 37 °C with continuous shaking at 30 rpm. Independent parallel samples were prepared for immersion periods of 1, 2, 3, 4, and 5 days.

At each time point, the supernatant from the fluoride-release experiment was collected, and the fluoride ion concentration was measured using a fluoride ion-selective electrode. In a separate parallel experiment conducted under the same immersion conditions, independent samples were prepared for each time point, and the pH of the surrounding medium was measured using a calibrated pH meter (SevenCompact S220, Mettler Toledo, Switzerland) to evaluate the effect of FPHA on medium pH.

Degradation-associated fluoride release was further evaluated under different pH conditions. Separate FPHA samples (250 mg each) were immersed in 5 mL of physiological buffer at pH 7.4, Tris–citric acid buffer at pH 3.0, or HCl–KCl buffer at pH 1.0. The samples were incubated at 37 °C with continuous shaking at 30 rpm. For the pH 7.4 and 3.0 conditions, independent samples were prepared for 1, 2, 3, 4, and 5 days. Because of the rapid degradation observed at pH 1.0, this condition was evaluated at 2, 6, 12, 24, 36 and 48 h. At each time point, fluoride ion concentration and degradation rate were determined. Supernatants collected at the final time point of each condition—day 5 for pH 7.4 and 3.0 and 48 h for pH 1.0—were additionally analyzed for calcium, phosphorus, and magnesium concentrations using inductively coupled plasma–optical emission spectrometry (ICP-OES; Agilent 720 ES, Agilent Technologies, USA).

### 2.5 Animal study design and surgical procedures

Twelve healthy male beagle dogs, aged 12–18 months and weighing 10–15 kg, with normal general condition, complete dentition, and no obvious oral or systemic disease, were included. Animals were excluded if they exhibited an abnormal general condition, oral or maxillofacial infection, incomplete dentition, mandibular anatomical abnormalities, abnormal baseline clinical findings, or any condition considered by the attending veterinarian to be unsuitable for surgery or long-term observation. All animal procedures were performed in accordance with protocols approved by the Institutional Animal Care and Use Committee of Sun Yat-sen University (SYSU-IACUC-2024-003090). This study is reported in accordance with the ARRIVE guidelines.

The sample size was justified using bone volume fraction (BV/TV) measured by micro-CT as the representative efficacy outcome. The FPHA-versus-DBBM comparison was selected as the primary sample-size-driving contrast because it represented the principal comparison between the investigational material and a clinically established xenogeneic bone substitute. Based on previously reported canine data, in which the BV/TV values of FPHA and DBBM were 24.77% ± 2.20% and 15.68% ± 3.15%, respectively, an expected difference of approximately 9 percentage points was adopted (Li et al., 2020). A simulation-based power assessment was performed using PASS 2026 version 26.0.1 (NCSS, LLC, USA) under a mixed-effects model reflecting the within-animal treatment structure. The simulation assumed a marginal variance of 6.93, a covariance of 2.31, and a within-animal correlation of approximately 0.33, based on the pooled variability reported in the previous canine study and the clustered within-animal treatment design. With a two-sided Bonferroni-adjusted α of 0.0167, a target power of 80%, and 5,000 simulations, four animals per time point provided an estimated power of approximately 82.5% for the FPHA-versus-DBBM comparison. Accordingly, 12 animals were included, with four animals assigned to each sacrifice time point.

Accordingly, the animals were randomly assigned to sacrifice at 4, 12, or 26 weeks after graft implantation, with four animals at each time point. A split-mouth design was used. To minimize potential interference between adjacent defects filled with different graft materials, FPHA and DBBM were placed on opposite mandibular sides. Within each animal, the mandibular side assigned to FPHA or DBBM was determined using a computer-generated random-number sequence. On the FPHA-assigned side, the mesiodistal positions of the two FPHA defects and the Blank defect were randomly allocated using the same procedure; the positions of the two DBBM defects and the Blank defect on the contralateral side were allocated in the same manner. Overall, each sacrifice group contained 24 defects, including eight FPHA, eight DBBM, and eight Blank defects.

Complete blinding of the outcome assessors was not feasible because the FPHA, DBBM, and Blank sites exhibited distinguishable radiographic and histological appearances. Nevertheless, standardized outcome-assessment procedures were consistently applied to reduce potential observer-related bias.

Before surgery, atropine at 0.05 mg/kg was administered intramuscularly 15 min before anesthesia induction. General anesthesia was induced by intramuscular injection of Sumianxin at 0.05 mL/kg and Zoletil at 0.2 mL/kg. The mandibular premolars and molars from P3 to M2 were carefully extracted bilaterally using a split-root technique.

After an 8-week healing period, three standardized box-shaped defects measuring 10 × 9 × 5 mm were created at the buccal aspect of each hemimandible using a carbide bur under continuous saline irrigation. Thus, six defects were created in each animal.

**FIGURE 1:**
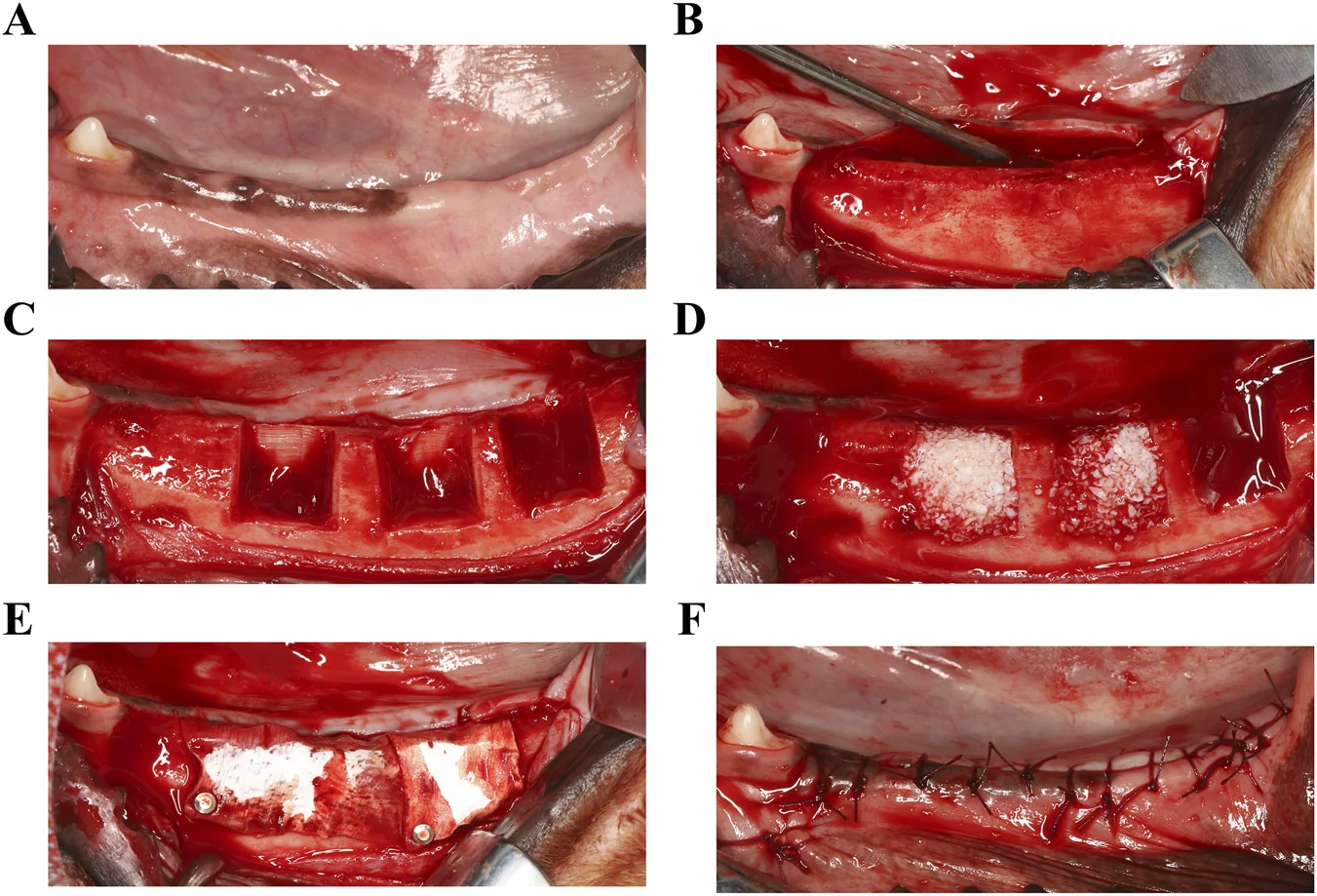
Surgical procedures of beagle dog mandibular defect model. **(A)** Preoperative view of the mandible 8 weeks after tooth extraction. **(B)** Exposure of the alveolar bone surface following incision and mucoperiosteal flap elevation. **(C)** Three standardized box-type defects (9 mm in height from the crestal bone, 5 mm in depth from the surface of the buccal bone, and 10 mm in width mesiodistally) were bilaterally created at the buccal aspect of each hemimandible (n = 6 defects per animal). **(D)** Augmentation treatment for each defect. Graft materials were applied to homogeneously fill the complete defect area. **(E)** All sites were protected by a collagen membrane covering 2 mm of the surrounding alveolar bone. **(F)** The mucoperiosteal flaps were advanced to ensure a tension-free submerged healing procedure.

All defect sites were covered with collagen membranes (Bio-Gide®, Geistlich, Switzerland), after which the flaps were repositioned and sutured. Analgin and penicillin sodium were administered intramuscularly, and cetylpyridinium chloride mouthwash was applied for 7 days postoperatively. The surgical procedure and defect allocation are illustrated in Figure 1.

Throughout the experimental period, the animals were fed a soft diet and monitored at least weekly for general condition, food intake, body weight, wound healing, pain or distress, infection, and other postoperative complications.

Humane endpoints were predefined according to the approved animal protocol. Humane intervention or early euthanasia would have been performed in the event of approximately 15%–20% body-weight loss, persistent anorexia, severe weakness, moribund appearance, cyanosis of the lips or oral mucosa, marked respiratory depression, inability to eat or drink independently, severe infection, tumor formation, or any other condition considered by the attending veterinarian to compromise animal welfare.

At the designated time points, the animals were deeply anesthetized and euthanized with an overdose of Zoletil. The mandibular specimens were harvested and fixed in 4% paraformaldehyde for 10 days.

### 2.6 Blood biochemical analysis

Venous blood samples were collected from the forelimb vein of each dog before graft implantation and immediately before euthanasia at its designated 4-, 12-, or 26-week endpoint. Serum fluoride concentration was measured using a fluoride ion meter, whereas the remaining biochemical parameters were measured using an automated biochemical analyzer (IDEXX Catalyst One®, IDEXX Laboratories, USA). The analyzed parameters included: (1) indicators of systemic metabolism and homeostasis, including serum fluoride (F), calcium (Ca), phosphorus (P), total protein (TP), albumin (ALB), globulin (GLOB), albumin/globulin ratio (ALB/GLOB), and cholesterol (CHOL); and (2) indicators of organ function and injury, including alanine aminotransferase (ALT), alkaline phosphatase (ALKP), gamma-glutamyl transferase (GGT), total bilirubin (TBIL), creatinine (CREA), urea (UREA), amylase (AMYL), and lipase (LIPA).

### 2.7 Micro-computed tomography (micro-CT) analysis

Each mandible was sectioned into six individual blocks, with each block containing one defect site. Micro-CT scanning was performed using a micro-computed tomography system (Pingseng Healthcare, Kunshan, China) with the following parameters: resolution 20 μm, voltage 80 kV, and current 200 μA. Before specimen scanning, a calibration phantom containing four inserts with known mineral densities was scanned under the same acquisition conditions. A calibration curve relating grayscale values to mineral density was established and applied to calculate trabecular bone mineral density (Tb.BMD) within the newly formed bone segment.

Three-dimensional images were reconstructed using Avatar software (Pingseng Healthcare, Kunshan, China). For each defect, the volume of interest (VOI) was defined according to the contour of the original surgically created defect. Adjacent native bone and other structures outside the original defect boundaries were excluded to minimize interference.

For image visualization, the window width and window level were consistently set at 6,000 and 4,000, respectively. Because FPHA and DBBM exhibited higher grayscale intensities than newly formed bone, a fixed grayscale threshold of 3,300 was consistently applied to distinguish newly formed bone from residual graft material. A consistent VOI definition and fixed segmentation criteria were applied to all specimens.

For contour analysis, the defect height (DH) was measured from the bottom of the defect to the highest point of the alveolar ridge, and the width of the augmented ridge (WR) was measured at 0%, 25%, 50%, 75%, and 100% of the defect height.

Residual material volume fraction (RMV/TV) was calculated from the residual graft-material segment to assess material resorption. The following parameters were calculated from the newly formed bone segment: bone volume fraction (BV/TV), trabecular thickness (Tb.Th), trabecular number (Tb.N), trabecular separation (Tb.Sp), and trabecular bone mineral density (Tb.BMD).

### 2.8 Histological observation

All specimens were dehydrated through a graded ethanol series and infiltrated and embedded in methyl methacrylate for undecalcified sectioning. After complete polymerization, serial sections were cut from the central region of each defect site in the buccolingual direction using an Exakt 300 CP cutting system (Exakt Apparatebau GmbH, Germany). The sections were ground to a final thickness of approximately 100 μm and stained with McNeal’s tetrachrome staining solution containing basic fuchsine and toluidine blue O. Histological observations were performed qualitatively under a light microscope to assess newly formed bone, residual graft material, and graft integration within the defect areas.

### 2.9 Statistical analysis

The statistical strategy was reviewed in consultation with a statistician from the School of Public Health, Sun Yat-sen University. Statistical methods were selected according to the experimental design and data structure of each dataset. Data normality was assessed using the Shapiro-Wilk test.

For the *in vitro* experiments involving more than two independent groups, one-way analysis of variance followed by Bonferroni-adjusted pairwise comparisons was used. For the degradation assay, FPHA and DBBM were compared within each pH condition using unpaired Student’s *t*-tests, with Bonferroni adjustment applied to the two pH-specific comparisons.

For the serum fluoride and blood biochemical analyses, the animal was regarded as the unit of analysis. Preoperative and postoperative measurements from the same animal were treated as paired observations and analyzed separately within the 4-, 12-, and 26-week groups. Paired *t*-tests were used when the paired differences were normally distributed; otherwise, Wilcoxon signed-rank tests were applied. Each biochemical parameter was regarded as a separate biological endpoint. For each parameter, Bonferroni correction was applied to the three time-point-specific preoperative-versus-postoperative comparisons.

For bone-regeneration and ridge-contour outcomes, linear mixed-effects models were used to evaluate the effects of treatment, time point, and defect location while accounting for the clustering of multiple defect sites within the same animal. Individual defect sites were treated as the observational units, whereas the animal was regarded as the biological experimental unit and included as a random intercept. Treatment, time point, mandibular side, and mesiodistal defect position were included as fixed effects. A treatment-by-time interaction term was also included to assess whether the treatment effect differed across time points. Bonferroni-adjusted pairwise comparisons of estimated marginal means were performed among the FPHA, DBBM, and Blank groups within each time point. For RMV/TV, pairwise comparisons were restricted to the FPHA and DBBM groups because no residual material was present in the Blank group. Ridge width measured at 0%, 25%, 50%, 75%, and 100% of the defect height was analyzed using separate mixed-effects models, with each measurement level treated as a distinct outcome variable.

Linear mixed-effects analyses were performed using IBM SPSS Statistics (IBM Corp., Armonk, NY, USA), and the remaining statistical analyses were performed using GraphPad Prism version 9.0 (GraphPad Software, USA). The simulation-based power assessment was performed using PASS 2026 version 26.0.1 (NCSS, LLC, USA). Descriptive data are presented as the mean ± standard deviation. Pairwise comparisons from the mixed-effects models were based on estimated marginal means, with Bonferroni adjustment for multiple comparisons. All statistical tests were two-sided, and a *p* value <0.05 was considered statistically significant.

## 3 Results and discussion

### 3.1 Fluoride was incorporated into the PHA lattice through fluoride–hydroxyl substitution

**FIGURE 2:**
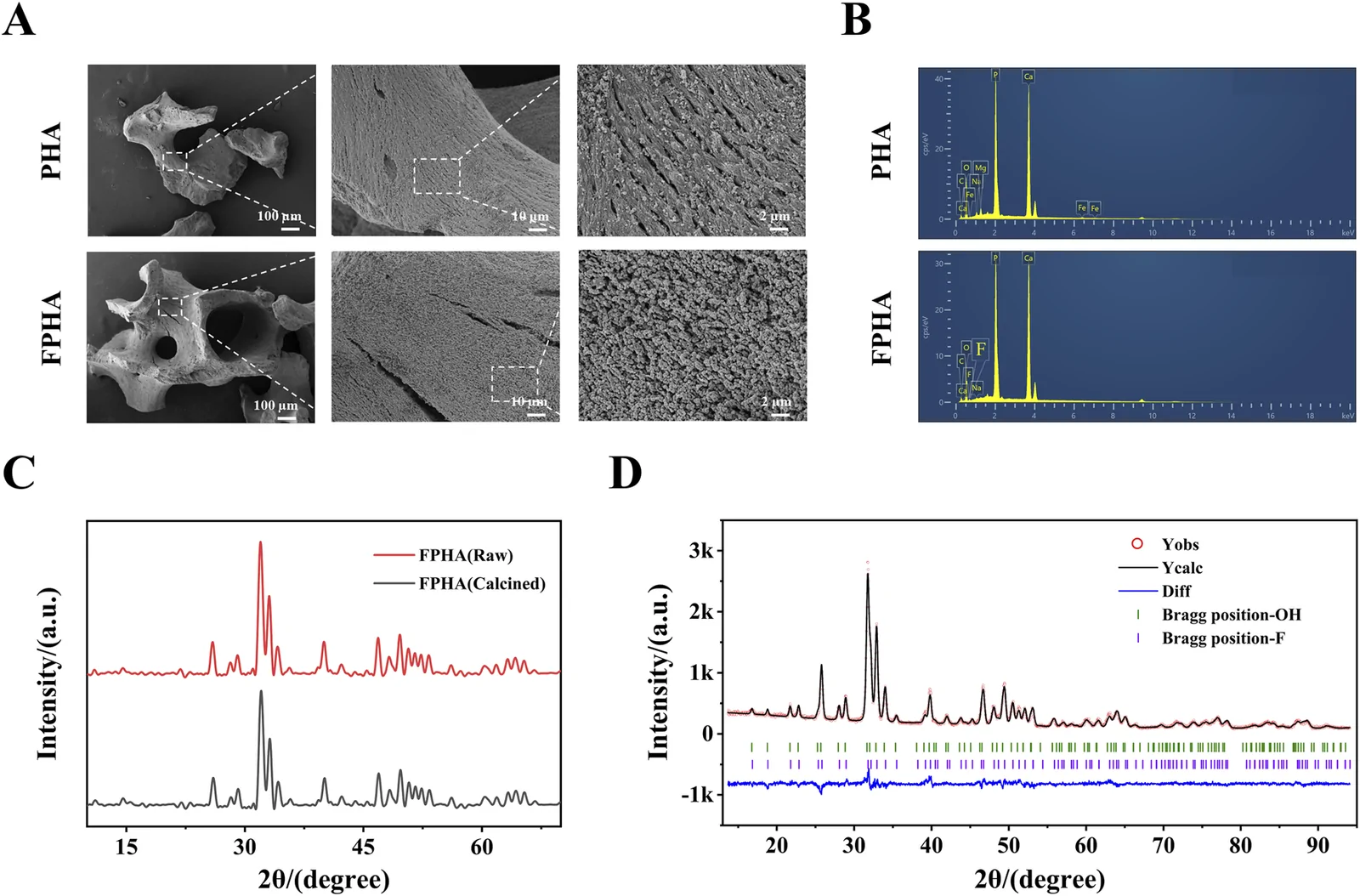
Characterization of PHA and FPHA. **(A)** SEM images showing the microscopic porous structure of PHA and FPHA. **(B)** EDS spectra confirming the elemental composition of PHA and FPHA. **(C)** XRD patterns of FPHA before and after calcination, used for crystallinity calculation. **(D)** Rietveld refinement of the FPHA XRD pattern for the determination of phase composition and unit-cell parameters.

Initially, qualitative structural assessments were conducted to verify the success of fluoride modification. Specifically, SEM observations revealed that FPHA retained the inherent three-dimensional biomimetic porous structure of natural bone; at the microscopic scale, FPHA exhibited a more regular and compact surface structure than unmodified PHA (Figure 2A). EDS analysis preliminarily confirmed the presence of fluoride in FPHA (Figure 2B). The EDS results indicated that both PHA and FPHA were primarily composed of carbon (C), oxygen (O), phosphorus (P), and calcium (Ca). Notably, the atomic percentage of fluorine on the FPHA surface was approximately 1.48% (Table 1), whereas no fluorine was detected in the unmodified PHA. XRD analysis elucidated the crystallographic characteristics of the materials. By evaluating the XRD peak areas, the crystallinity of FPHA was calculated to be over 99% (Figure 2C; Table 2). Rietveld refinement analysis was performed using TOPAS software to determine the crystal structure and phase composition of FPHA (Figure 2D) (De Figueiredo and Ferreira, 2014). The results demonstrated that the fluorination process preserved the characteristic hexagonal symmetry of hydroxyapatite while yielding a composite phase comprising 65.56 wt% fluorapatite and 34.44 wt% hydroxyapatite (Table 3), supporting the successful substitution of hydroxyl groups by fluoride ions.

**TABLE 1:** Quantitative elemental composition of PHA and FPHA surfaces determined by EDS analysis (At%).

​	At (%)					
Material	C	O	F	Na	P	Ca
PHA	14.84	48.99	—	0.66	12.74	21.59
FPHA	14.91	47.07	1.48	0.21	12.19	24.14

**TABLE 2:** Crystallinity assessment of FPHA based on XRD peak area analysis.

Sample type	Peak 1	Peak 2	Peak 3	Peak 4	Peak 5	Peak 6	Peak 7	Peak 8	Peak 9	Peak 10	Total integrated area	Crystallinity (%)
FPHA(Raw)	371	142	238	1302	467	919	318	367	430	433	4,896	99.98
FPHA(Calcined)	418	137	271	1303	522	853	307	344	387	446	4,987	

**TABLE 3:** Rietveld refinement results of FPHA, including phase identification, unit cell parameters, and phase content.

Parameter	Phase 1: Ca_5_(PO_4_)_3_F (Fluorapatite)	Phase 2: Ca_5_(PO_4_)_3_OH (Hydroxyapatite)
Space group	P6_3_/m (no. 176)	P6_3_/m (no. 176)
Unit cell parameters		
a (Å)	9.4092 (16)	9.4477 (19)
b (Å)	9.4092 (16)	9.4477 (19)
c (Å)	6.8900 (13)	6.9263 (16)
α (°)	90	90
β (°)	90	90
γ (°)	120	120
Unit cell volume (Å^3^)	528.26 (21)	535.41 (25)
Phase content (wt%)	65.56	34.44

These results were consistent with our previous findings. At the micromorphological scale, the surface structure of FPHA appeared more regular and denser than that of unmodified PHA (Qiao et al., 2017). Zhang et al. and Arca-Garcia et al. also reported that fluoride incorporation increased the density of hydroxyapatite microstructures, which may contribute to improved microstructural integrity and antibacterial properties (Zhang et al., 2024; Arca-Garcia et al., 2026). Owing to the smaller ionic radius of fluoride (F^−^) compared with that of hydroxyl (OH^−^), this isomorphous substitution resulted in a reduced unit-cell volume (Bertoni et al., 1998), contributing to a more compact crystal arrangement and potentially improving the degradation stability of FPHA.

In summary, these characterization findings support the lattice-bound incorporation of fluoride ions into FPHA, providing a structural basis for the subsequent dissolution-based estimation of the total fluoride content.

### 3.2 Determination of the TFC of FPHA using a dual-validation dissolution approach

Reliable estimation of the total fluoride content (TFC) is important for the preclinical evaluation and potential clinical translation of FPHA. A dual-validation dissolution approach was therefore employed to obtain cross-validated estimates of the TFC.

**FIGURE 3:**
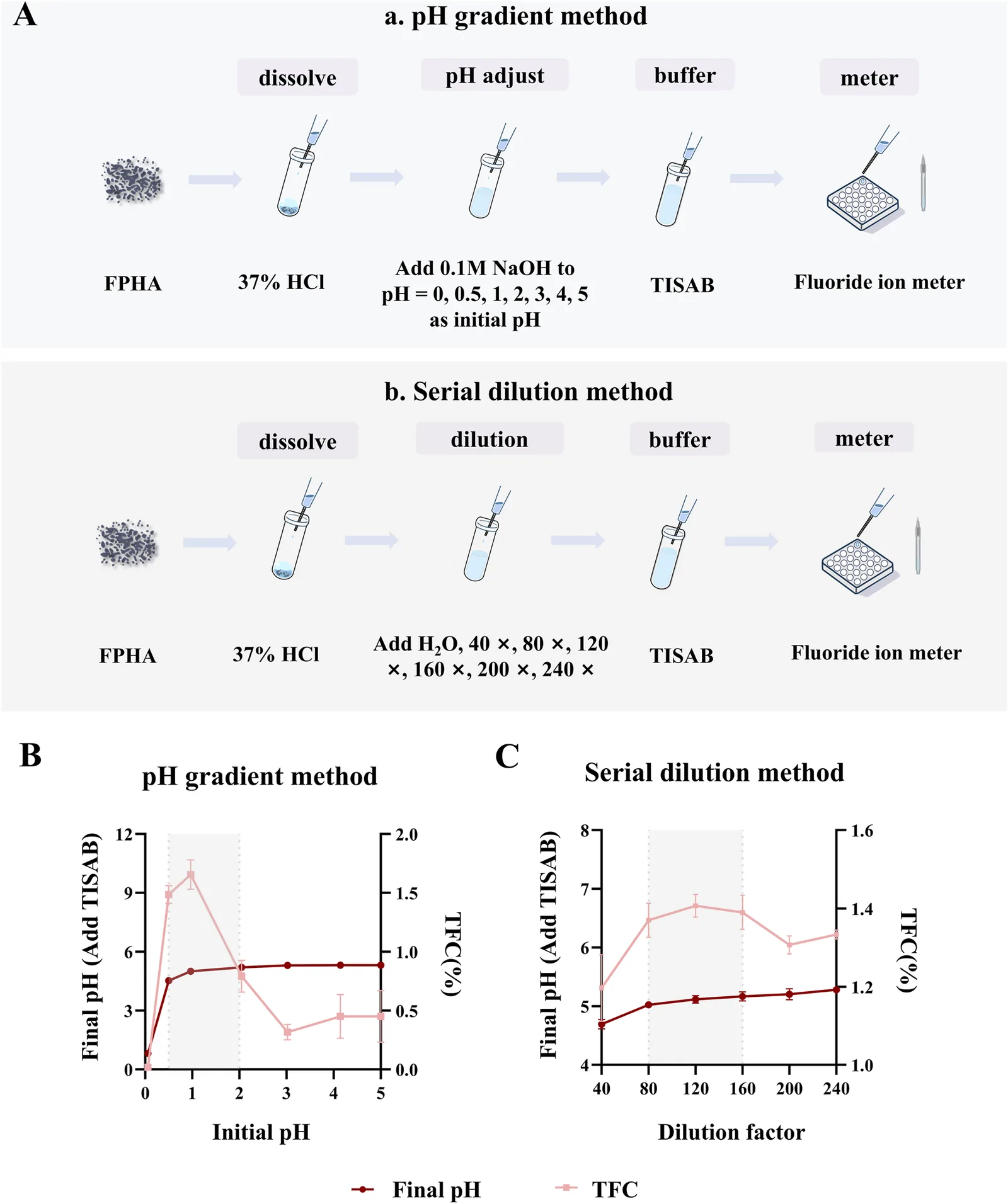
Quantitative analysis of the total fluoride content (TFC) in FPHA. **(A)** Schematic illustration of the dual-validation methodology, including **(a)** pH gradient method and **(b)** serial dilution method. **(B)** Final pH values (post-TISAB buffer addition) and calculated TFC across different initial target pH levels. **(C)** Final pH values (post-TISAB buffer addition) and calculated TFC across different dilution factors. Data are presented as means ± SD.

The general strategy for determining the TFC involved completely dissolving FPHA in HCl and measuring the fluoride concentration using a fluoride ion-selective electrode (ISE). Given that the fluoride ISE requires an appropriate pH range for reliable measurement, strongly acidic conditions may promote the formation of undissociated hydrofluoric acid (HF), whereas excessive alkalinization may induce secondary precipitation of calcium fluoride (CaF_2_) (Ogaard, 2001). Both conditions may interfere with the reliable detection of free fluoride ions. Therefore, a pH-gradient method was established to identify an appropriate detection window (Figures 3A,a). The measured fluoride concentration was highest when the initial pH of the dissolved sample was between 0.5 and 2.0, with the highest calculated TFC reaching approximately 1.66% under the selected pH-adjustment condition (Figure 3B; Table 4).

**TABLE 4:** Determination of total fluoride content in FPHA by pH gradient method.

Target pH	Final pH (TISAB buffered)	F^−^ concentration (mg/L)	Total fluoride content (%)
0 (0.07 ± 0.05)	0.81 ± 0.23	0.7 ± 0.7	0.022 ± 0.020
0.5 (0.50 ± 0.11)	4.52 ± 0.09	49.6 ± 2.5	1.487 ± 0.076
1 (0.97 ± 0.08)	5.00 ± 0.08	55.2 ± 4.1	1.656 ± 0.123
2 (2.05 ± 0.08)	5.21 ± 0.04	33.2 ± 13.5	0.992 ± 0.403
3 (3.02 ± 0.02)	5.30 ± 0.02	10.6 ± 2.2	0.319 ± 0.065
4 (4.14 ± 0.10)	5.31 ± 0.00	13.2 ± 3.5	0.397 ± 0.104
5 (5.00 ± 0.04)	5.31 ± 0.01	13.1 ± 4.6	0.393 ± 0.137

Data are presented as means ± SD.

To reduce potential interference associated with NaOH-mediated pH adjustment, including hydroxide interference and possible secondary precipitation, a serial-dilution method was employed for cross-validation (Figures 3A,b). Serial dilution progressively reduced the acidity of the strongly acidic solution without direct alkali addition. Although the measured fluoride concentration decreased with increasing dilution, the calculated TFC reached a maximum at the 120-fold dilution, corresponding to approximately 1.40% (Figure 3C; Table 5). The two complementary methods yielded maximum calculated TFC values of approximately 1.66% and 1.40%, respectively. Based on these cross-validated estimates, the bulk TFC of FPHA was estimated to be approximately 1.40%–1.66%.

**TABLE 5:** Determination of total fluoride content in FPHA by serial dilution method.

Dilution factor	(Initial pH)	Final pH (TISAB buffered)	F^−^ concentration (mg/L)	Total fluoride content (%)
40×	(0.85 ± 0.04)	4.69 ± 0.08	29.9 ± 2.2	1.196 ± 0.088
80×	(1.04 ± 0.03)	5.02 ± 0.05	17.1 ± 0.5	1.368 ± 0.040
120×	(1.25 ± 0.01)	5.12 ± 0.07	11.7 ± 0.2	1.404 ± 0.024
160×	(1.36 ± 0.06)	5.17 ± 0.08	8.7 ± 0.3	1.392 ± 0.048
200×	(1.46 ± 0.10)	5.21 ± 0.09	6.5 ± 0.1	1.300 ± 0.020
240×	(1.56 ± 0.07)	5.28 ± 0.02	5.6 ± 0.1	1.344 ± 0.024

Data are presented as means ± SD.

The fluorine signal detected by EDS and the TFC estimated using the dissolution-based methods provided complementary evidence for the presence of fluoride in FPHA at both the surface and bulk levels. Compared with surface-sensitive techniques such as EDS, dissolution-based analysis provides complementary information on the bulk fluoride content and is not restricted to near-surface probing depth (Newbury and Ritchie, 2013). Acid dissolution followed by ISE detection is an established approach for apatite analysis (McCann, 1968). However, fixed neutralization protocols require careful control to minimize the formation of undissociated HF under acidic conditions and secondary precipitation during excessive alkalinization (Petzold, 2001). In the present study, pH-gradient optimization was used to identify an appropriate measurement condition, whereas serial-dilution analysis provided an alternative approach for cross-validating the estimated TFC without direct alkali addition. Cross-validation between the two methods increased confidence in the estimated bulk TFC of FPHA. However, because method-specific LOD, LOQ, recovery, precision, matrix effects, and spike-recovery were not independently established, the resulting values should be interpreted as cross-validated estimates rather than as fully validated precise measurements. This combined approach may provide a useful methodological reference for estimating fluoride content in fluoride-containing calcium phosphate materials, although its broader applicability requires further analytical validation.

### 3.3 FPHA exhibits a low degradation rate and controlled fluoride release under physiological and artificial extreme conditions

**FIGURE 4:**
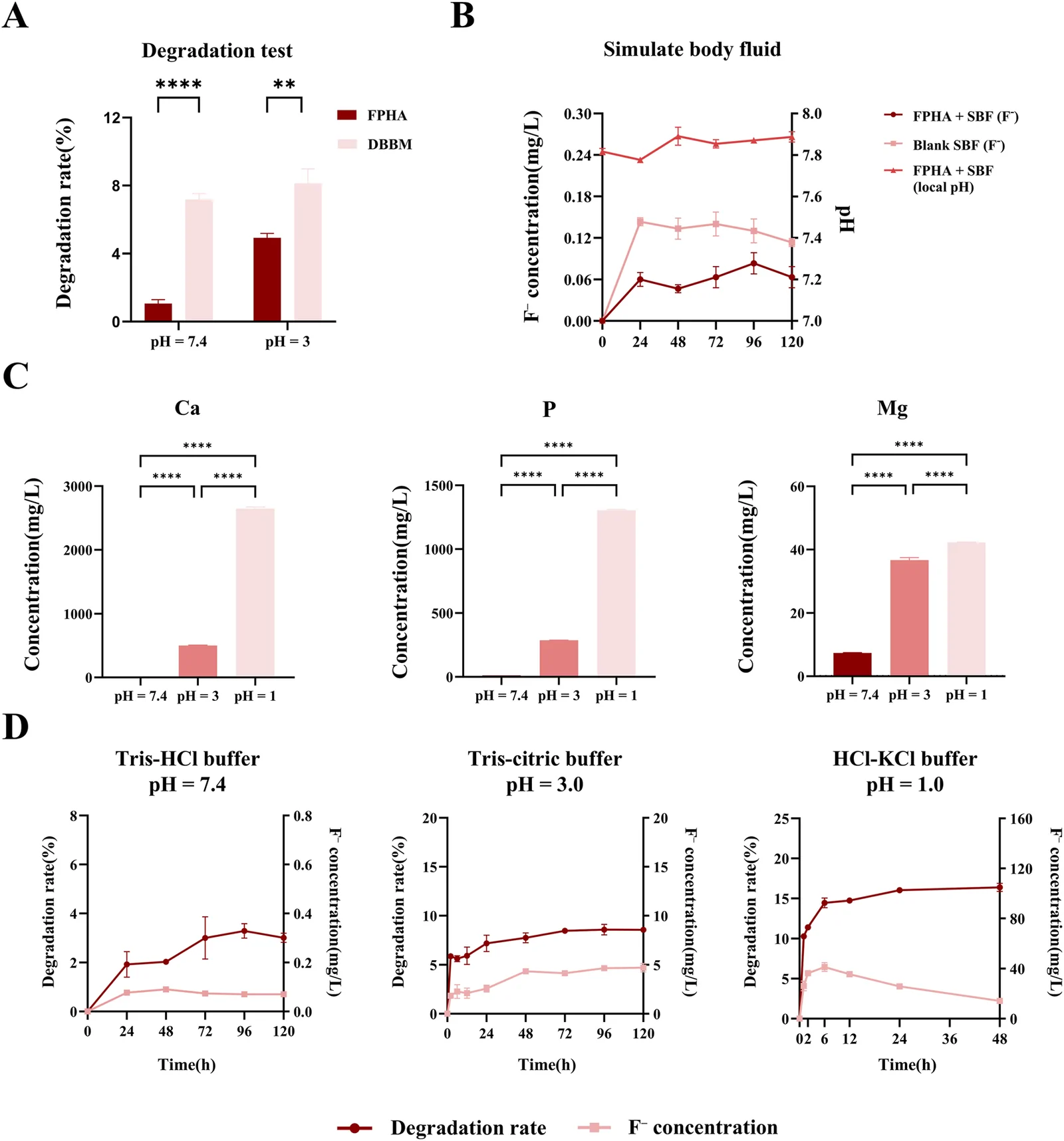
Evaluation of *in vitro* degradation behaviors and fluoride release profiles of FPHA. **(A)** Degradation rates of FPHA and DBBM under physiological (pH 7.4) and artificial extreme (pH 3.0) conditions. FPHA and DBBM were compared separately within each pH condition using unpaired Student’s *t*-tests, with Bonferroni correction applied to the two pH-specific comparisons. **(B)** Free fluoride concentration and pH of the surrounding medium of FPHA in simulated body fluid (SBF). **(C)** Calcium (Ca), phosphorus (P), and magnesium (Mg) concentrations in the supernatants collected at the condition-specific final time points—day 5 for pH 7.4 and 3.0 and 48 h for pH 1.0 were measured by inductively coupled plasma–optical emission spectrometry (ICP-OES). For each ion, comparisons among the pH conditions were performed using one-way analysis of variance followed by Bonferroni-adjusted pairwise comparisons. **(D)** Degradation rate and degradation-associated fluoride release of FPHA under physiological (pH 7.4) and artificial extreme (pH 3.0 and 1.0) conditions. Data are presented as the mean ± SD (n = 3, *p < 0.05, **p < 0.01, ***p < 0.001, ****p < 0.0001).

The degradation behavior of a bone graft material is relevant to its ability to maintain space for bone regeneration. Two *in vitro* dissolution conditions were established: a physiological model at pH 7.4 and an artificial extreme model at pH 3.0. FPHA showed a significantly lower degradation rate than DBBM under both conditions (Figure 4A), indicating greater chemical stability *in vitro*. Fluoride release was subsequently evaluated using an SBF immersion model under simulated physiological conditions and pH-controlled degradation models. FPHA released less than 0.1 mg/L free fluoride throughout the observation period under simulated physiological conditions (Figure 4B). Using parallel samples under the same SBF immersion conditions, the pH of the surrounding medium remained within a narrow range of 7.8–7.9 throughout the 5-day immersion period, with no marked fluctuation observed (Figure 4B).

To further characterize degradation-associated fluoride release, FPHA was evaluated in pH-controlled models at pH 7.4, 3.0, and 1.0. The pH 7.4 condition was used as a physiological model, whereas pH 3.0 and pH 1.0 were used as artificial extreme models for material stability testing. ICP-OES results showed that the concentrations of Ca, P, and Mg increased significantly as the pH decreased (*p* < 0.0001), consistent with increased material dissolution under more acidic conditions (Figure 4C). The fluoride-release profiles at pH 7.4 and 3.0 gradually approached a plateau over time, whereas the fluoride concentration at pH 1.0 showed an initial peak followed by a decline (Figure 4D). This biphasic pattern may reflect rapid initial surface dissolution followed by changes in fluoride speciation and/or secondary precipitation as the solution chemistry evolved.

The lower degradation rate of FPHA may be related to the reduced solubility of hydroxyapatite following fluoride incorporation (Qiao et al., 2017). Under simulated physiological conditions, the concentration of free fluoride released from FPHA remained below 0.1 mg/L, which was substantially lower than the reported *in vitro* cytotoxicity threshold of 10 mg/L for osteoblasts (Wu et al., 2019), supporting a controlled fluoride-release profile under the tested conditions.

In the pH-controlled degradation models, the fluoride concentrations reached approximately 0.5, 5, and 40 mg/L at pH 7.4, 3.0, and 1.0, respectively. As a very rough worst-case estimate, assuming instantaneous and complete transfer of the fluoride released from the 5-mL experimental system followed by homogeneous dilution in 4 L of blood, the theoretical concentration increments corresponding to the fluoride concentrations measured at pH 7.4, 3.0, and 1.0 would be approximately 6.25 × 10^−4^, 6.25 × 10^−3^, and 5.0 × 10^−2^ mg/L, respectively. Even the highest estimated increment was below the reported human chronic toxicity threshold of 0.069 mg/L and acute renal toxic limit of 0.76 mg/L (Allison et al., 2021). However, this simplified calculation does not account for the actual implanted material mass, material surface area, local microenvironment, time-dependent release, tissue distribution, metabolic clearance, renal excretion, or species differences. Therefore, it should be regarded only as a very rough worst-case estimate rather than a prediction of actual systemic fluoride exposure *in vivo*.

### 3.4 Blood-based assessments support a favorable systemic biosafety profile of FPHA over 26 weeks *in vivo*

Systemic safety is an important consideration for the clinical translation of bone substitutes. As bone regeneration and material integration generally approach a relatively stable stage by 6 months in large animal models, a 26-week follow-up period was employed to evaluate the systemic response associated with FPHA implantation in the beagle dog model. Throughout their respective observation periods, all animals remained in good general health. Clinical observations revealed that FPHA achieved favorable soft tissue integration; the surgical-site mucosa remained healthy, exhibiting no clinical signs of erythema, edema, or persistent inflammatory exudation (Supplementary Figures S1–S3).

**FIGURE 5:**
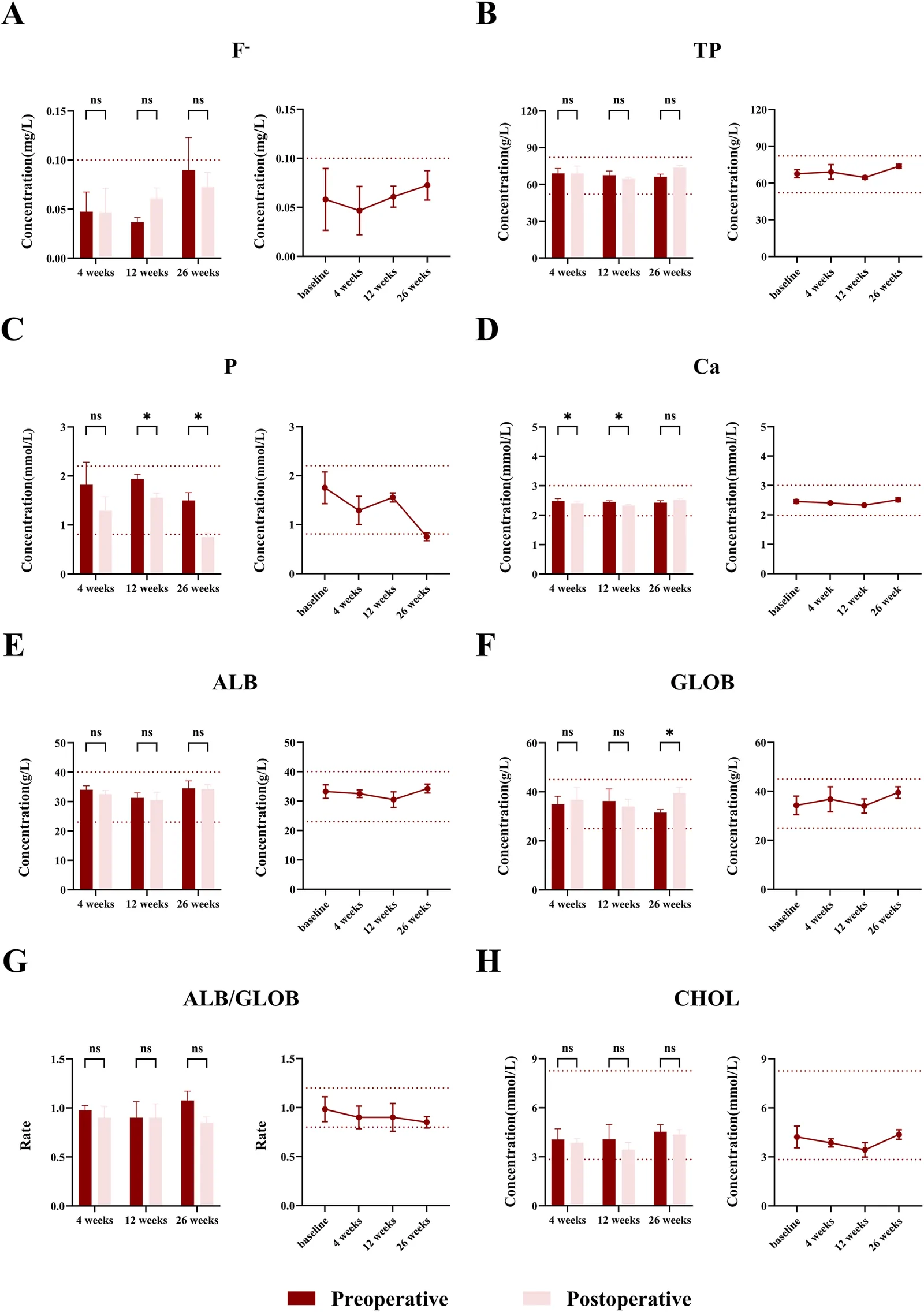
Serum fluoride concentrations and systemic metabolic indicators in beagle dogs during the animal study. **(A)** Serum fluoride concentration. **(B–H)** Blood biochemical indicators including total protein (TP), phosphorus (P), calcium (Ca), albumin (ALB), globulin (GLOB), ALB/GLOB ratio, and cholesterol (CHOL). Preoperative and postoperative values were compared separately within the 4-, 12-, and 26-week groups using paired Student’s *t*-tests when the paired differences were normally distributed and Wilcoxon signed-rank tests otherwise. Bonferroni correction was applied across the three time-point-specific comparisons for each parameter. Data are presented as the mean ± SD. (*p < 0.05, **p < 0.01, ***p < 0.001, ****p < 0.0001).

Serum fluoride concentrations were assessed before implantation and at the designated postoperative endpoint in each time-point group. As shown in Figure 5A, no statistically significant differences were detected between the preoperative baseline and the corresponding postoperative endpoint within the 4-, 12-, or 26-week groups. Across all samples, serum fluoride concentrations ranged from approximately 0.04–0.10 mg/L, with no apparent postoperative increase relative to the corresponding preoperative baseline.

**FIGURE 6:**
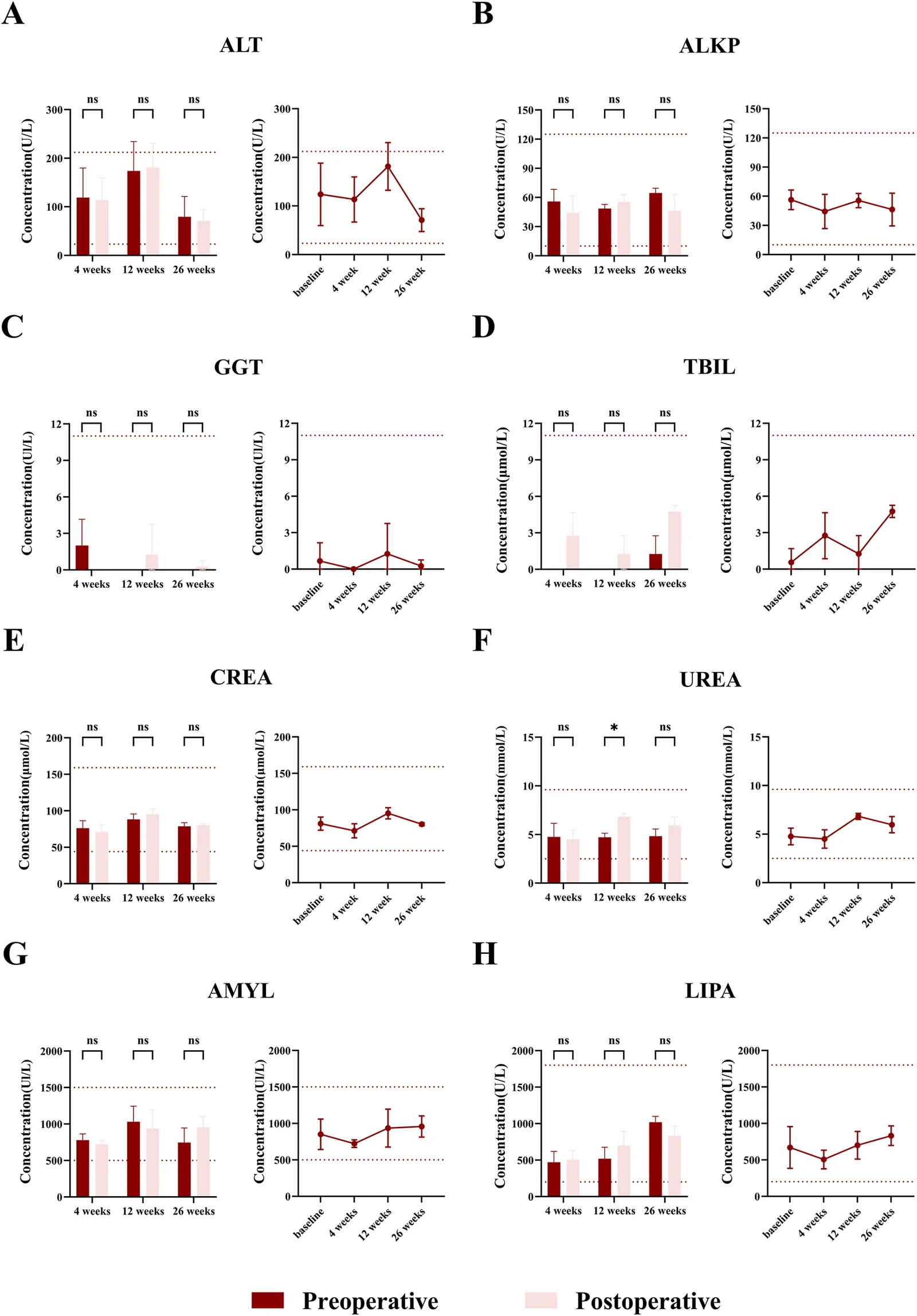
Blood biochemical indicators related to organ function in beagle dogs during the animal study. **(A–D)** Liver-related biochemical parameters: alanine aminotransferase (ALT), alkaline phosphatase (ALKP), gamma-glutamyl transferase (GGT), and total bilirubin (TBIL). **(E, F)** Kidney-related biochemical parameters: creatinine (CREA) and urea (UREA). **(G, H)** Pancreatic enzymes: amylase (AMYL) and lipase (LIPA). Preoperative and postoperative values were compared separately within the 4-, 12-, and 26-week groups using paired Student’s *t*-tests when the paired differences were normally distributed and Wilcoxon signed-rank tests otherwise. Bonferroni correction was applied across the three time-point-specific comparisons for each parameter. Data are presented as the mean ± SD. Each time-point group comprised four animals. (*p < 0.05, **p < 0.01, ***p < 0.001, ****p < 0.0001).

Blood biochemical analyses provided complementary information regarding systemic metabolic status and parameters associated with hepatic, renal, and pancreatic function (Figures 5, 6). Most parameters showed no statistically significant changes relative to their corresponding preoperative baselines and generally remained within the laboratory reference intervals. A small number of parameters exhibited statistically significant fluctuations, including a marginal decrease in serum phosphorus at 26 weeks. However, these changes were not accompanied by consistent abnormalities in related biochemical indicators or by clinically apparent adverse responses, and no coherent biochemical pattern suggestive of systemic metabolic or major-organ dysfunction was identified.

Published reference data on serum fluoride concentrations in dogs remain limited, particularly for animals matched by breed, sex, age, and body weight. A previous experimental study reported a mean preanesthetic serum fluoride concentration of 4.54 ± 0.82 μmol/L in dogs (Pedersoli, 1977), corresponding to approximately 0.086 ± 0.016 mg/L. The 0.04–0.10 mg/L range observed in the present study was broadly comparable to the former value. However, given the differences in breed, sex, age, body weight, dietary fluoride exposure, sample type, analytical methods, and experimental conditions, this reported value should be regarded only as a contextual reference rather than as a physiological range in the present study. Therefore, the present findings were interpreted primarily according to changes from the individual preoperative baseline rather than against a predefined physiological reference range. Because each animal simultaneously received FPHA, DBBM, and Blank treatments, the systemic measurements reflected the overall response to the combined surgical and material interventions rather than the isolated systemic effect of FPHA. Moreover, major-organ histopathological examination was not performed; therefore, localized or subclinical tissue alterations that were not reflected by the blood-based parameters cannot be excluded. Within these limitations, the blood-based findings provide preliminary evidence supporting a favorable systemic biosafety profile of FPHA over the 26-week observation period in the present preclinical model.

### 3.5 FPHA exhibits effective ridge contour maintenance and enhanced bone-regenerative performance

**FIGURE 7:**
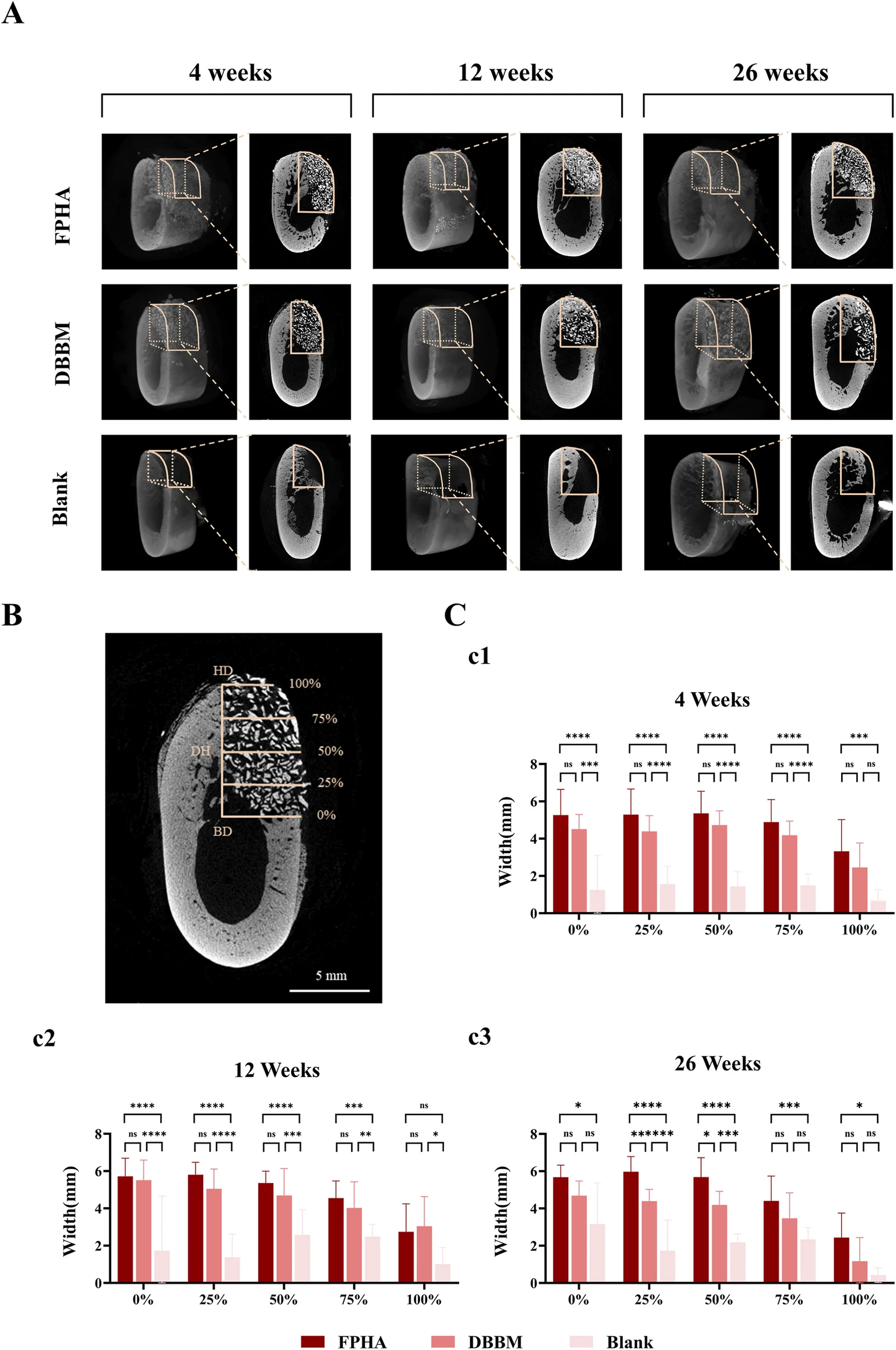
Representative micro-CT images and micro-CT-based ridge contour analysis. **(A)** Representative three-dimensional and cross-sectional micro-CT reconstructions of beagle mandibular defects at 4, 12, and 26 weeks after graft implantation. **(B)** Schematic illustration of ridge contour analysis. The following landmarks were identified in the micro-CT image: the bottom of the defect (BD) and the highest point of the alveolar ridge of the defect (HD). The defect height (DH, mm) was measured from the HD to the BD, and the width of the augmented ridge (WR, mm) was measured at 0%, 25%, 50%, 75%, and 100% of the DH. **(C) (c1–c3)** Quantitative analysis of the horizontal ridge width at five horizontal levels (0%, 25%, 50%, 75%, and 100% of the defect height) at **(c1)** 4 weeks, **(c2)** 12 weeks, and **(c3)** 26 weeks. Each predefined height level was analyzed as a separate outcome using a linear mixed-effects model, with animal included as a random effect and treatment, time, mandibular side, mesiodistal defect position, and the treatment-by-time interaction included as fixed effects. Bonferroni-adjusted pairwise comparisons of estimated marginal means were performed among the FPHA, DBBM, and Blank groups within each time point. Data are presented as the mean ± SD. The animal was considered the biological experimental unit, and each treatment-by-time group comprised eight defect-level observations from four animals. (*p < 0.05, **p < 0.01, ***p < 0.001, ****p < 0.0001).

The bone-regenerative performance of the materials was further evaluated by micro-CT analysis. Representative three-dimensional and cross-sectional reconstructions demonstrated progressive bone remodeling and gradual graft material degradation throughout the experimental period (Figure 7A). Both FPHA and DBBM effectively maintained the defect contour, with graft particles and newly formed bone filling the defect area, whereas the collapse of the defect contour was observed in the blank group. Qualitatively, FPHA appeared to show broad graft distribution and mineralized tissue formation within the defect region.

To quantitatively evaluate ridge contour maintenance, horizontal ridge width at each predefined height level (0%, 25%, 50%, 75%, and 100%) was analyzed as a separate outcome using linear mixed-effects models (Figure 7B). A significant treatment effect was detected at all five levels (all *p* < 0.001), whereas time, mandibular side, mesiodistal defect position, and the treatment-by-time interaction were not significant at any level. At 4 and 12 weeks, FPHA and DBBM showed comparable ridge widths at all measured levels and generally exhibited greater widths than the Blank group at the 0%–75% levels (Figure 7C,c1, 2). At 26 weeks, FPHA exhibited greater ridge widths than the Blank group at all five levels and greater widths than DBBM at the 25% and 50% levels (*p* = 0.005 and *p* = 0.017, respectively; Figure 7C,c3). No significant differences between FPHA and DBBM were detected at the remaining levels. Both FPHA and DBBM provided effective structural support for the box-shaped defects, as reflected by the generally greater ridge widths compared with the Blank group. At 26 weeks, FPHA showed greater widths than DBBM at the 25% and 50% levels, suggesting favorable contour preservation in the middle portion of the defect.

**FIGURE 8:**
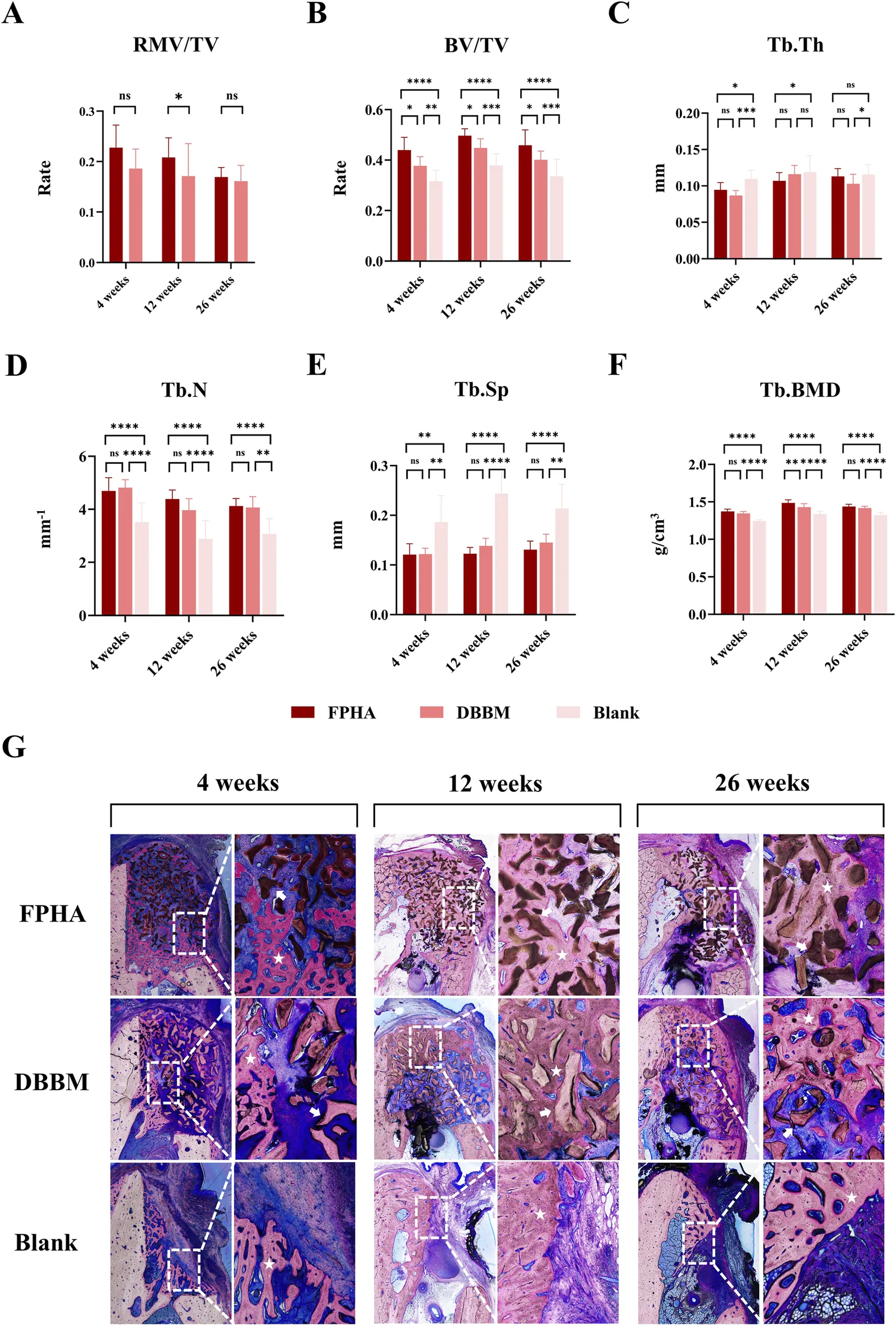
Micro-CT-based quantitative analysis and representative histological images. **(A–F)** Quantitative evaluation of residual graft material and newly formed bone within the defect region: **(A)** residual material volume fraction (RMV/TV), **(B)** bone volume fraction (BV/TV), **(C)** trabecular thickness (Tb.Th), **(D)** trabecular number (Tb.N), **(E)** trabecular separation (Tb.Sp), and **(F)** trabecular bone mineral density (Tb.BMD). **(G)** Representative histological images of the FPHA, DBBM and Blank groups at 4, 12, and 26 weeks post-implantation. White asterisks, new bone; white arrows, residual material. Each micro-CT outcome was analyzed separately using a linear mixed-effects model, with animal included as a random effect and treatment, time, mandibular side, mesiodistal defect position, and the treatment-by-time interaction included as fixed effects. For RMV/TV, only the FPHA and DBBM groups were included in the analysis. Bonferroni-adjusted pairwise comparisons of estimated marginal means were performed among the relevant treatment groups within each time point. Data are presented as the mean ± SD. The animal was considered the biological experimental unit, and each treatment-by-time group comprised eight defect-level observations from four animals. (*p < 0.05, **p < 0.01, ***p < 0.001, ****p < 0.0001).

Residual material volume fraction was evaluated to characterize graft persistence within the defect region. The linear mixed-effects model revealed a significant effect of time on RMV/TV (*p* = 0.012), whereas the main effect of material and the material-by-time interaction were not significant (both *p* > 0.05). FPHA exhibited a higher RMV/TV than DBBM at 12 weeks (*p* = 0.015), while the two materials showed comparable RMV/TV values at 4 and 26 weeks (*p* > 0.05, Figure 8A). Although FPHA showed a higher RMV/TV at 12 weeks, the absence of differences at 4 and 26 weeks does not support a consistently slower degradation profile than DBBM.

Bone formation and trabecular architecture were subsequently evaluated using micro-CT-derived morphometric parameters. Linear mixed-effects analyses revealed significant treatment effects on BV/TV, Tb.N, Tb.Sp, and Tb.BMD (all *p* < 0.0001), as well as on Tb.Th (*p* = 0.00022). Significant overall time effects were detected for Tb.Th, Tb.N, and Tb.BMD, whereas no significant time effects were observed for BV/TV or Tb.Sp. No treatment-by-time interactions or defect-location effects were detected for these outcomes. Bonferroni-adjusted pairwise comparisons showed that FPHA exhibited significantly higher BV/TV than DBBM at 4, 12, and 26 weeks (*p* = 0.029, *p* = 0.033, and *p* = 0.024, respectively; Figure 8B). Both FPHA and DBBM exhibited higher BV/TV than the Blank group at all three time points (Figure 8B). No significant differences between FPHA and DBBM were detected in Tb.Th at any time point. Compared with the Blank group, FPHA exhibited higher Tb.Th at 4 and 12 weeks, whereas DBBM exhibited higher Tb.Th at 4 and 26 weeks (Figure 8C). Similarly, FPHA and DBBM showed comparable Tb.N and Tb.Sp throughout the observation period. Both grafted groups exhibited higher Tb.N and lower Tb.Sp than the Blank group at all three time points (Figures 8D,E). Tb.BMD was higher in both grafted groups than in the Blank group at all time points. FPHA and DBBM showed comparable Tb.BMD at 4 and 26 weeks, whereas FPHA exhibited significantly higher Tb.BMD than DBBM at 12 weeks (*p* = 0.002; Figure 8F). The most consistent difference between FPHA and DBBM was observed in BV/TV, which was higher in the FPHA group at all three time points. This finding indicates that FPHA was associated with a greater volume of micro-CT-defined newly formed bone under the present experimental conditions. Both graft materials also exhibited higher Tb.N, lower Tb.Sp, and higher Tb.BMD than the Blank group, indicating the formation of a denser and more highly mineralized trabecular network within grafted defects. The absence of significant differences in Tb.Th, Tb.N, and Tb.Sp between FPHA and DBBM suggests broadly comparable trabecular architecture between the two groups. The higher Tb.BMD observed with FPHA at 12 weeks represents a time-specific difference and should not be interpreted as a persistent advantage throughout healing.

Overall time comparisons indicated that Tb.Th was higher at 12 weeks than at 4 weeks (*p* = 0.045), Tb.N was lower at both 12 and 26 weeks than at 4 weeks (*p* = 0.042 and 0.036, respectively), and Tb. BMD was higher at both 12 and 26 weeks than at 4 weeks (both *p* < 0.001). The temporal changes in the trabecular parameters were consistent with ongoing bone remodeling. The lower Tb.N at 12 and 26 weeks, together with the higher Tb.Th at 12 weeks and higher Tb. BMD at 12 and 26 weeks, may reflect ongoing remodeling from an initially fine trabecular network toward a more highly mineralized trabecular structure (Kustro et al., 2018; Collier et al., 2020).

Qualitative histological observations of non-decalcified ground sections were generally consistent with the micro-CT findings. At 4 weeks, both the FPHA and DBBM groups maintained the alveolar ridge contour, whereas the Blank group showed apparent buccal contour collapse. Newly formed bone extended mainly from the adjacent marrow spaces and appeared broadly distributed in the FPHA group (white asterisks; Figure 8G). At 12 weeks, newly formed bone extended through much of the FPHA-filled defects and was observed in close contact with the graft particles (white arrows; Figure 8G). In the DBBM group, newly formed bone appeared more concentrated in the upper and middle portions of the defect. The Blank group showed limited bone ingrowth from the alveolar crest, together with contour collapse in the middle and lower regions. At 26 weeks, FPHA-filled defects showed preserved contour and broadly distributed newly formed bone relative to the Blank group (Figure 8G).

Collectively, the micro-CT and qualitative histological findings indicate that FPHA provided effective ridge-contour maintenance and supported bone formation within the augmented defects. In this study, quantitative histomorphometry and mechanistic analyses were not performed. These osteogenic findings should be interpreted as observational evidence based primarily on the quantitative micro-CT results.

### 3.6 Future perspectives and limitations

The present study illustrates a fluoride-specific, biosafety-oriented translational pathway that complements conventional biomaterial assessment. Conventional preclinical evaluation generally includes material characterization, degradation analysis, biocompatibility assessment, and *in vivo* efficacy testing. Given the dose-dependent biological effects of fluoride, additional fluoride-specific assessments were incorporated into the evaluation of FPHA: for material characterization, two complementary dissolution-based methods were used to obtain cross-validated estimates of the total fluoride content; for degradation analysis, physiological and artificial extreme models were established to assess material degradation, fluoride release, and pH changes of the surrounding medium; for biocompatibility assessment, a 26-week beagle dog study including serum fluoride monitoring, blood biochemical analyses, and *in vivo* assessment of material persistence was conducted. This study not only provides preliminary evidence to support the translational pathway of FPHA, but also offers a reference for the preclinical evaluation and translation of similar ion-functionalized biomaterials.

Several limitations should be acknowledged. Mechanistic experiments explaining the bone formation observed with FPHA were not performed. Previous studies demonstrated that FPHA may modulate the osteoimmune microenvironment and promote bone regeneration (Chen et al., 2024). Future studies will elucidate the mechanisms underlying FPHA-associated osteogenesis using direct-contact culture models with FPHA. Because each animal simultaneously received FPHA, DBBM, and Blank treatments, serum fluoride concentrations and blood biochemical parameters reflected the overall condition of the animals following the combined surgical and material interventions rather than the isolated systemic effect of FPHA. Therefore, the safety findings should be regarded as preliminary blood-based evidence.

## 4 Conclusion

A fluoride-specific, biosafety-oriented translational pathway was developed for the preclinical evaluation of FPHA. FPHA exhibited a favorable preliminary biosafety profile, effective ridge contour maintenance, and enhanced bone formation compared to deproteinized bovine bone mineral.

## Data Availability

The raw data supporting the conclusions of this article will be made available by the authors, without undue reservation.
