# Supplementary material for: Integrating biosafety and osteogenic efficacy: a translational pathway of fluorinated porcine-derived hydroxyapatite for bone regeneration
Source: Front Bioeng Biotechnol. 2026 Aug 25;14:1889739. doi: 10.3389/fbioe.2026.1889739 (PMC13550175; doi:10.3389/fbioe.2026.1889739)
Supplement: Supplementary file 1 [file Supplementaryfile1.docx]

**Supplementary material**

**Supplementary Figure 1. The peri-implant mucosa of beagle dogs 4 weeks following implantation**

**Supplementary Figure 2. The peri-implant mucosa of beagle dogs 12 weeks following implantation**

**Supplementary Figure 3. The peri-implant mucosa of beagle dogs 26 weeks following implantation**
